# Traction-separation law parameters for the description of age-related changes in the delamination strength of the human descending thoracic aorta

**DOI:** 10.1007/s10237-024-01871-1

**Published:** 2024-07-10

**Authors:** Zdeněk Petřivý, Lukáš Horný, Petr Tichý

**Affiliations:** Faculty of Mechanical Engineering, Czech Technical University in Prague, Technická 4, 160 00 Prague, Czech Republic

**Keywords:** Aging, Cohesive model, Crack, Damage, Finite elements method, Fracture

## Abstract

Aortic dissection is a life-threatening disease that consists in the development of a tear in the wall of the aorta. The initial tear propagates as a discontinuity leading to separation within the aortic wall, which can result in the creation of a so-called false lumen. A fatal threat occurs if the rupture extends through the whole thickness of the aortic wall, as blood may then leak. It is generally accepted that the dissection, which can sometime extend along the entire length of the aorta, propagates via a delamination mechanism. The aim of the present paper is to provide experimentally validated parameters of a mathematical model for the description of the wall’s cohesion. A model of the peeling experiment was built in Abaqus. The delamination interface was described by a piecewise linear traction-separation law. The bulk behavior of the aorta was assumed to be nonlinearly elastic, anisotropic, and incompressible. Our simulations resulted in estimates of the material parameters for the traction-separation law of the human descending thoracic aorta, which were obtained by minimizing the differences between the FEM predictions and the delamination force given by the regression of the peeling experiments. The results show that the stress at damage initiation, *T*_*c*_, should be understood as an age-dependent quantity, and under the assumptions of our model this dependence can be expressed by linear regression as *Tc* =  − 13.03·10^−4^·Age + 0.2485 if the crack front advances in the axial direction, and *Tc* =  − 7.58·10^−4^·Age + 0.1897 if the crack front advances in the direction of the aortic circumference (*T*_*c*_ [MPa], Age [years]). Other model parameters were the stiffness *K* and the separation at failure, δ_*f*_–δ_*c*_ (*K* = 0.5 MPa/mm, δ_*f*_–δ_*c*_ = 0.1 mm). The material parameters provided by our study can be used in numerical simulations of the biomechanics of dissection propagation through the aorta especially when age-associated phenomena are studied.

## Introduction

The aorta is the largest artery in the human body and its main function is to take up all the blood ejected by the left ventricle and to conduct it to the branches leading to all parts of the body (Kassab 2007; Wang et al. 2023). Since, the heart beats ceaselessly, the aorta faces challenging mechanical conditions at which it is under continual loading. Although its anatomical structure makes the aorta well prepared to perform its function, there are diseases that threaten its mechanical integrity and can lead to its failure (Thubrikar 2007). One of these diseases is aortic dissection, which consists in delamination of the tissue of the aortic wall so that a crack spreads along the axis and circumference of the aorta (Thubrikar 2007; Tong et al. 2016; Sherifova and Holzapfel 2019, 2020; Amabili et al. 2020; Wang et al. 2023).

From a mechanical point of view, dissection develops in a number of typical stages (Sherifova and Holzapfel 2019, 2020). First, the onset of the dissection that occurs when an initial tear is formed on the inner side of the aortic wall in the layer referred to as tunica intima. As blood enters the arterial wall, the tear spreads radially and a delamination process subsequently begins, typically in the middle layer of the artery referred to as the tunica media. The structure of the media is lamellar, and during the delamination one lamella separates from the other, causing the crack to spread further in the circumferential and axial directions, and new crack tips are formed. This mechanism may result in the formation of a new lumen which, if loaded with blood pressure, will not spontaneously close because the crack changes the local pattern of the radial stress (MacLean et al. 1999). Finally, one or more of the following complications may occur: (1) blood flowing in newly created (false) lumen can cause a blockage or a complete closure of an artery that branches out of the aorta leading to a decreased perfusion in the terminal tissue, or (2) the crack tip can propagate through the weakened aortic wall in a radial direction to the tunica adventitia until it reaches the outer surface of the aorta. The first case is dangerous, especially due to the possible blockage of the main branches of the coronary arteries (Prêtre and Von Segesser 1997), while the second means that blood starts to leak out of the aorta (Tanaka et al 2009).

The delamination mechanism described above suggests a key role for the radial integrity of the arterial wall. The organization of the internal structure itself has been studied by Wolinsky and Glagov (1967) and Clark and Glagov (1985), who described in detail the musculo-elastic fascicles that define the lamellar structure of the media. Radial mechanical properties that reflect interlamellar cohesion were studied in a direct tension test by MacLean et al. (1999), Sommer et al. (2008), and Tong et al. (2011). It was concluded that the strength in the radial direction is significantly lower than the strength in circumferential and axial direction (MacLean et al. 1999).

An important series of experiments focused on the cohesion of the arterial wall was carried out by M. R. Roach and her colleagues. They studied dissection propagation in experiments which were based on pumping a pressurized liquid into the blood vessel wall (van Baardwijk and Roach 1987; Carson and Roach 1990; Tiessen and Roach 1993). It was revealed that pressure gradient, rather than peak pressure, is correlated with the dissecting, which is in accordance with the results of Prokop et al. (1970). It was also found that dissections propagated more easily between the elastic lamellae than across them.

**Fig. 1:**
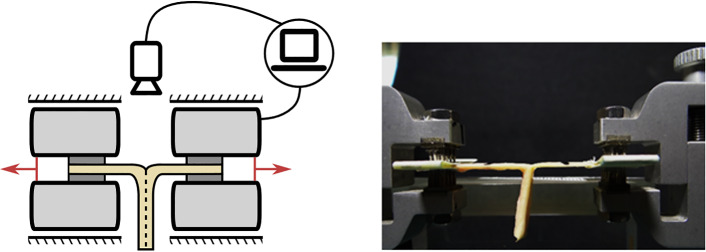
Peeling experiment configuration

In the current biomechanical literature, there is a growing number of papers investigating delamination strength using the peeling experiment (Sommer et al. 2008; Tong et al. 2011, 2014; Pasta et al. 2012; Kozuń 2016; Kozuń et al. 2018; Myneni et al. 2020; Horný et al. 2022). The peeling experiment consists of creating a T-shaped specimen from the strip of an artery, which is incised in its thickness so as to achieve delamination by pulling on the arms of the T specimen (Fig. 1). In the experiment, the force required to advance the crack tip along the artery strip is measured and the delamination strength is then defined as this force divided by the width of the specimen. The experimental procedure resembles mode I crack opening and the fracture energy can be obtained in this way.

It has been found that delamination strength is a site-specific property (Tong et al. 2011; Myneni et al. 2020; Horný et al. 2022; Sokolis and Papadodima 2022a, b; Ríos-Ruiz et al. 2022) which is in accordance with previous results obtained by Roach and Song (1994). It has also been found that delamination resistance is anisotropic, because longitudinally oriented strips usually exhibit a higher delamination strength than circumferential strips (Sommer et al. 2008; Tong et al. 2011; Kozuń et al. 2016; Horný et al. 2022). Studies by Pasta et al. (2012), Angouras et al. (2019), Chung et al. (2020), Kozuń et al. (2018), and Tong et al. (2023) have shown that pathologies like ascending aortic aneurysm or atherosclerosis are associated with a decreased delamination resistance of the aorta.

Significant attention has also recently been paid to the mathematical modeling of cohesive properties and to the numerical simulations that reproduce the propagation of dissection or delamination through the arterial wall (Gasser and Holzapfel 2003, 2006; Ferrara and Pandolfi 2010; Pal et al. 2014; Wang et al. 2018, 2021a; Noble et al. 2017). The studies differ in the approaches used to introduce crack propagation. Some of them work with XFEM (eXtended Finite Element Method), where crack can propagate in any direction, but others instead use predefined interfaces through which cracks are spread. These interfaces can be based on cohesive zone (CZ) elements or on contact bond models. The fact that the location of crack propagation is known in advance is advantageously used in computational analyses of peeling experiments (Leng et al. 2015, 2016; Merei et al. 2017; Miao et al. 2020; Ríos-Ruiz et al. 2021; Yu et al. 2020; Wang et al. 2021a,b).

However, compared to the studies that report peeling experiment results, there are still few studies that identify the parameters for the mathematical models of cohesion. Gasser and Holzapfel (2006) identified the parameters of the cohesive model suitable for the FEM modeling of one representative of the human aortic media, and the same experimental data were used in Ferrara and Pandolfi (2010). Pal et al. (2014) developed an analytical model which described cohesive failure based on the idea of a disruption of radially running collagen fibers (Tsamis et al. 2013) and demonstrated its calibration on data collected in peeling experiments with the human ascending thoracic aorta (Pasta et al. 2012). Leng and colleagues determined the parameters for describing the cohesive zone model for the delamination of atherosclerotic plaque (Leng et al. 2015, mouse model; Leng et al. 2016, human tissue). Leng et al. (2018) and Ríos-Ruiz et al. (2021) investigated the differences in the delamination behavior associated with an experimental protocol. Wang et al. (2021a) identified the traction–separation law parameters for the porcine aortic media and investigated the effect of the elastin network. In order to reduce the number of parameters identified by means of FEM simulations, they used the statistical interpretation of oscillations on the force–displacement signal to determine the separation distance at damage initiation. The same concept was used in Wang et al. (2021b) in the investigation of the effect of glycation on the interlamellar bonding properties of arterial elastin. In very recent work, Donahue et al. (2024) have also employed a combination of delamination force and its oscillations to reveal the effect of atherosclerosis on the cohesive model parameters.

As shown above, many studies identify the parameters of the mathematical models for cohesive behavior with the use of animal samples instead of human tissue. This, however, limits the applicability of the predictions made by these models. This is best understood when one considers the typical length of a human life, within which aging-associated changes in physiology develop, and compares it to the typical lifespan of an animal model (decades vs. months). When studying age-related phenomena, the use of human tissues seems to be unavoidable. To the best of our knowledge, the current literature lacks experimentally validated material parameters for describing human aortic cohesion which would account for aging. Their estimation based on the use of the FEM model of the peeling experiment is the main objective of the present study.

## Methods

**Fig. 2:**
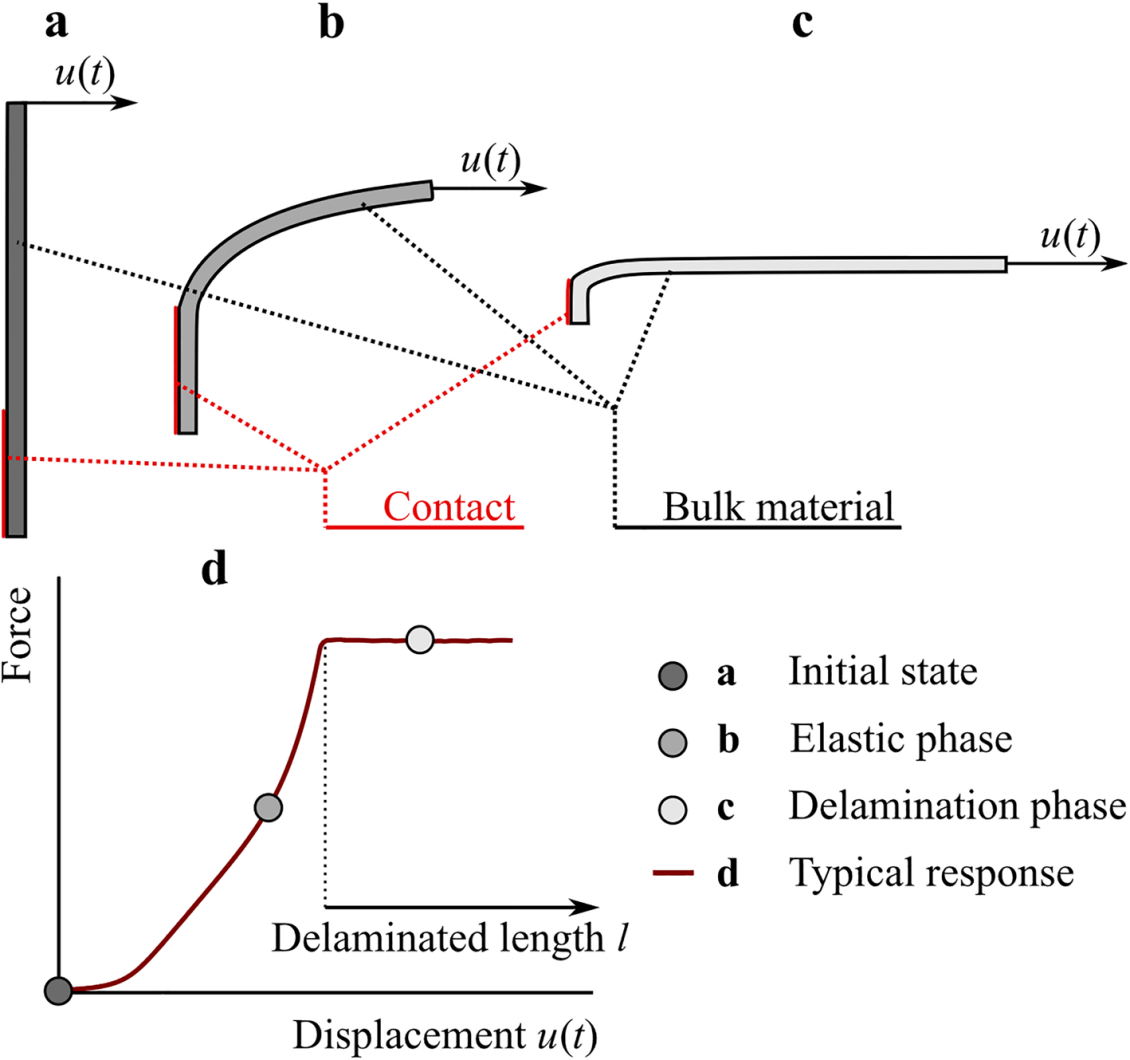
Schema of the peeling test simulation. The plane symmetry was utilized within the building of the FEM model. Panels **a**–**c** depict 3 phases of the mechanical response of the T-shaped specimen to kinematical loading in the peeling test. The length of the cohesive interface in the elastic phase (**b**) is the same as in the initial state (**a**); the cohesive interface is denoted as “Contact”. When the displacement, *u* = *u*(*t*), assigned to the end of the strip induces a traction that exceeds cohesion, the delamination occurs and the contact length begins to decrease. Panel **d** depicts idealized force–displacement relationship measured in the peeling experiment

The main objective of our study is to obtain experimentally validated estimates of the constitutive parameters for a model of cohesion at the delamination interface of the human descending thoracic aorta that would reflect age-related changes in aortic biomechanics. The displacements and forces measured during the peeling experiment play a role of observation in our study. The material parameters for the linear traction–separation law, expressing mathematically the delamination behavior, will be calibrated against the measured delamination force using the finite element method (FEM) model of the peeling experiment. Figure 2 shows the phases that the FEM model goes through during the simulation of the peeling experiment. The model was built as 3D and the computational complexity was reduced by introducing the plane symmetry of the problem (cf. Figs. 1 and 2). During loading, the FEM model undergoes a phase of purely elastic behavior (bending and stretching) that eventually transitions to the separation phase, which is imposed by kinematical loading set as displacement *u* at the end of the T-shaped specimen. The details of this procedure are expressed in the following paragraphs.

### Elastic behavior

Under certain conditions (the elimination of history dependence, the irrelevance of dissipative processes, negligible rate-dependent phenomena) the arterial wall response can be considered (pseudo)elastic (Fung 1993). In such a case, the essential attributes of the mechanical response of the artery are considered to be its nonlinearity and anisotropy. Many models for the strain energy density function *W* can be found in the current literature (Fung et al. 1979; Takamizawa and Hayashi 1987; Holzapfel et al. 2000, 2015; Gasser et al. 2006; Labrosse et al. 2013; Horný et al. 2014a; Weisbecker et al. 2015). One of the most common phenomenological models, which fits well with known observations, while allowing for a microscopic interpretation of its parameters, is the model introduced in Holzapfel et al. (2000). It was used in our study. As a result, bulk aortic material was considered to be hyperelastic, anisotropic and incompressible. The specific expression for the strain energy density function used is given by (1).

1 $$W = \frac{\mu }{2}\left( {I_1 - 3} \right) + \frac{k_1 }{{2k_2 }}\sum_{j = 4,6} {\left( {e^{k_2 \left( {I_j - 1} \right)^2 } - 1} \right)}$$

In (1) *µ* and *k*_1_ are stress-like material parameters and *k*_2_ is dimensionless. *I*_1_ is the first principle invariant of the right Cauchy-Green deformation tensor, **C** which is obtained from the deformation gradient **F** as **C** = **F**^T^**F**. The Neo-Hooke term in (1) is assumed to be associated with the strain energy stored in the isotropic part of the extracellular matrix and smooth muscle cells, whereas the exponential term is assumed to account for the energy stored within the deformation of a network of collagen fibers that are accepted to be a cause of the anisotropic behavior of the aortic wall. In our specific case, the anisotropy is introduced by the existence of two preferred directions that are given by unit vectors ***M***_1_ and ***M***_2_ that lie in the $\Theta$-*Z* cylindrical plane of the blood vessel. Vectors ***M***_1_ and ***M***_2_ are considered to be placed symmetrically with respect to the $\Theta$ axis by an angle ± *β*. The invariants *I*_4_ and *I*_6_ are given by following equations: *I*_4_ = ***M***_1_·**C*M***_1_, and *I*_6_ = ***M***_2_·**C*M***_2_. More details about this model can be found in Holzapfel et al. (2000), Gasser et al. (2006), or Holzapfel and Ogden (2010). Readers interested in more information on the construction of additional invariants can refer to Spencer (1982), Holzapfel (2000), Itskov (2019), or, for example, Truesdell and Noll (2004).

### Cohesive behavior

Since, our model simulates crack propagation in the peeling experiment, it means that the crack propagation location is known a priori and there is no need to introduce advanced concepts like XFEM and similar techniques (Belytschko and Black 1999; Mohammadi 2008; Belytschko et al. 2009; Gasser and Holzapfel 2003; Wells et al. 2002; Wang et al. 2018). An approach referred to as *cohesive contact* was used to simulate aortic wall cohesion (Abaqus 2019). In this approach, the contact area represents the interface at which the cohesion of the artery wall fails (Fig. 2). In Abaqus, a traction-separation (T-S) law can be assigned to the contact area, including the conditions of damage initiation and damage evolution.

The T-S law represents the constitutive equation for cohesive behavior and brings together traction (stress) and separation (displacement) vectors at the cohesive interface. In our study, the piecewise linear T-S law was employed (Foresell and Gasser 2011; Merei et al. 2017; Ríos-Ruiz et al. 2021; Wang et al. 2021a,b; Donahue et al. 2024). In a 1D description, its elastic part is expressed in (2). Here, *T* represents the nominal traction in the normal direction, *δ* is the separation, and *K* represents the stiffness of the bond.

2 $$T = K\delta$$

**Fig. 3:**
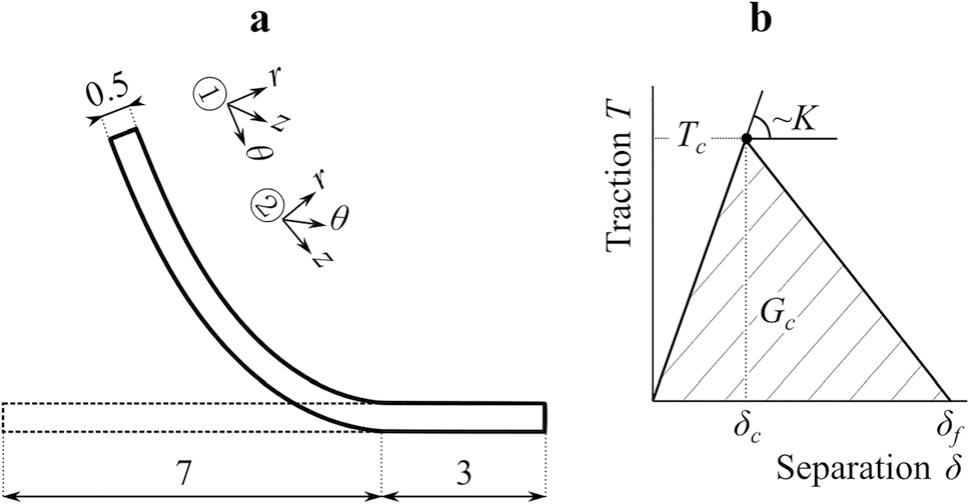
Panel **a** depicts the geometry of the model. Note, that the coordinate systems indicate two orientations of the delaminated specimen. Indicated dimensions are in mm. Panel **b** illustrates the T-S law. Here, *K* denotes the stiffness of the cohesion bond, *T*_*c*_ and *δ*_*c*_ are critical values of the traction vector and separation that correspond to the damage initiation, and *δ*_*f*_ is the maximal separation at which the bond fails and a new crack tip is formed

Figure 3b shows the graph explaining the function of the T-S law. *T* first increases linearly up to the value of *T*_*c*_. *T*_*c*_ is the critical value at which damage initiation occurs. As the damage develops, the T-S law graph decreases which in Fig. 3b is shown by the line segment connecting [*δ*_*c*_,*T*_*c*_] and [*δ*_*f*_,0]. Finally, when the separation displacement *δ* reaches the value of *δ*_*f*_, the contact bond fails, carried stress falls to zero and permanent separation occurs.

The size of the area of the triangle given in Fig. 3b by the vertices [0, 0], [*δ*_*c*_,*T*_*c*_], [*δ*_*f*_,0] represents the areal density of fracture energy *G*_*c*_. From the above, it is clear that in the 1D case, the T-S law can be expressed in terms of five parameters, *K*, *T*_*c*_, *δ*_*c*_, *δ*_*f*_, and *G*_*c*_, but only three of them are independent of each other.

### Finite element model, loading and boundary conditions

The software Abaqus (version 2019, Dassault Systèmes) was used to create the numerical model and to perform the simulations (Abaqus/Standard, inertial effects not considered). The model was built as 3D and plane symmetry was used to reduce the complexity of the problem. Figures 2 and 3a show the geometry and the position of the delamination interface created with the help of the contact area, where T-S law was assigned. The overall dimensions of the model were 10 mm × 0.5 mm (length × thickness of the strip) and the width was set to the width of one element. Let us add that the term thickness here is used in the same way as when talking about the thickness of a blood vessel (the in-plane dimensions in Fig. 3 are length and thickness, and width is an out-of-plane dimension).

Since, our study is based on using the FEM model to predict the delamination force, which is then compared with experiments, a sensitivity study of the results on the density of the mesh was performed. Meshes with element edge lengths of 0.1, 0.05, 0.025, and 0.00125 mm were used for FEM calculations in order to verify the mesh independence of the results. The final results, which will be shown below, were obtained with the mesh with an element edge length of 0.0125 mm, which corresponds to the total number of 371 657 C3D4H elements (hybrid linear tetrahedrons).

A loading was applied by means of a kinematic boundary condition. A displacement *u*(*t*) perpendicular to the length of the delaminated strip was prescribed at the end of the strip (see Fig. 2). During the delamination phase, the displacement perpendicular to the delamination interface corresponds to the length by which the delamination interface is shortened (Fig. 2). In order to ensure the stability of the geometry during loading, the width of the model was prescribed to be constant within the loading (Gasser and Holzapfel 2006; and Ferrara and Pandolfi 2010).

### Calibration of T-S law parameters

The aim of our study was to estimate the material parameters of the T-S law, which will be validated against the measured delamination forces. As described above, the FEM simulation of the peeling experiment was used as a regression analysis tool. The specific delamination force values to which the material parameters were fitted were adopted from our previous study Horný et al. (2022). In Horný et al. (2022), a total of 661 delamination experiments were performed with human aorta samples obtained from 46 cadavers. Here, we will limit ourselves to the results obtained for the descending thoracic aorta, as it represents one of the most dangerous sites for dissection propagation (it corresponds to type B according to the Stanford classification, Thubrikar 2007). Horný et al. (2022) conducted a total of 246 peeling experiments with samples obtained from the descending part of the thoracic aorta. The main conclusions yielded in their work are (1) cohesive properties are location-specific, (2) they are relatively independent of the extension rate (in the range 0.1–50 mms^−1^), (3) the delamination strength depends on the direction in which the crack tip propagates, and (4) the delamination strength depends on age.

Horný et al. (2022) presented their results in the form of graphs of experimental data points fitted with the regression model, expressing age-related changes in the delamination force in a mathematical form. Since, we want to study the age dependence of the T-S law parameters, it would be more useful to use the predictions of the delamination strength obtained by this linear regression, instead of working with each individual experimental observation (of which there are 122 for the longitudinal direction and 124 for the circumferential direction in Horny et al. (2022)). The results of the regression of *F*/*w* (delamination force per width) versus age found in Horný et al. (2022) are shown in Table 1. They are supplemented by confidence intervals for the regression line parameters and the variability of the observations.

**Table 1:** Regression equations and confidence intervals (CI) determined for age-related changes in the delamination strength of the human descendent thoracic aorta adopted to our study from Horný et al. (2022)

Orientation	*F*/*w* = *a*·Age + *b*	95%CI for *a*	95%CI for *b*	*R* *p*-value	Mean ± *SD*
*L*	− 3.1E−4·Age + 5.1E−2	± 1.3E−4	± 7.5E−3	− 0.41 *p* < E−5	3.3E−2 ± 1.1 E−2
*C*	− 1.9E−4·Age + 3.6E−2	± 8.6E−5	± 5.2E−3	− 0.36 *p* < E−4	2.5E−2 ± 7.6E−3

A classical linear model was used. *R* denotes correlation coefficient

At this point it is necessary to specify for which age values the calibration FEM simulations were performed. The individual ages were chosen in accordance with the constitutive description adopted for the bulk material. The material parameters presented in Jadidi et al. (2020), who reported the results of mechanical tests performed with human thoracic descending aorta samples obtained from 78 donors, were used. Table 2 shows both the ages (mean value and the age interval which the mean value represents) for which our FEM simulations were performed and the specific numerical values of the parameters for *W* expressed by (1).

**Table 2:** Age-related HGO model parameters of the bulk description of the descending thoracic aorta from (Jadidi et al. 2020)

Age [year]	*μ* [kPa]	${k}_{1}$[kPa]	${k}_{2}$[−]	$\beta$[°]
15.3 (13–20)	41.69	1.20	2.56	33.82
24.4 (21–30)	41.82	1.78	2.61	35.54
36.0 (31–40)	40.32	0.16	6.58	61.34
46.5 (41–50)	44.94	0.24	9.33	64.20
54.9 (51–60)	49.91	0.22	16.17	65.35
66.5 (61–70)	51.13	0.34	17.83	63.63
73.2 (71–78)	51.68	0.51	27.99	60.76

The elastic properties of the aorta, as well as its delamination strength, are anisotropic. This property is shared by model (1) and its numerical characterization in Jadidi et al. (2020), as well as by the equation for age-related changes in the delamination strength adopted from Horný et al. (2022). In the version of Abaqus used (Abaqus 2019), however, it was not possible to define a cohesive contact with an anisotropic T-S law. For this reason, simulations were split into three cases. The first one represents the delamination of an axially oriented strip (the crack face advances axially) calibrated against the delamination force obtained from an identically arranged experiment. The second case represents the same but for the circumferential direction (the circumferential strip and the delamination strength measured for it). The third case represents the calibration against the delamination force averaged from the axial and circumferential experiments. In this averaged case, the T-S law parameters were tuned so that the difference between the average of the force predicted by the simulation done with axial and circumferential strip and the average calculated form force measured in the axial and circumferential peeling test is minimal. The third case was included in our study mainly due to the fact that there are situations when it is not known in advance in which direction the crack will propagate, or what is the exact orientation of the specimen to the cylindrical coordinate system of the artery.

As shown above, the T-S model has a total of five parameters (*K*, *T*_*c*_, *δ*_*c*_, *δ*_*f*_, and *G*_*c*_), only three of which are independent. After pilot simulations aimed at finding conditions under which the FEM problem would be stable and converge well, it was decided to choose the value of *K* fixed at *K* = 0.5 MPa/mm. The same applies to the characterization of the separation. Abaqus operates with the quantity *δ*_*f*_ –*δ*_*c*_, which was held constant at *δ*_*f*_–*δ*_*c*_ = 0.1 mm. This left *T*_*c*_ as the only free parameter, facilitating the search for the minimum of the difference between the *F*/*w* (delamination strength) known from the regression of the experiments and that predicted by the FEM calculation. The optimization itself was controlled by manually specifying the value of *T*_*c*_ before running Abaqus.

## Results of the simulations: estimated T-S law parameters

A total of 21 FEM analyses were performed that represented the search for the optimal value of *T*_*c*_ based on the minimization of the differences between the measured delamination strength and the force predicted by FEM (3 orientations × 7 age points). The typical number of iterations (FEM simulations) after which an accepted difference between the delamination force from the experiment and from the FEM calculation was achieved, was 8 for the longitudinal and circumferential orientations and 14 for the averaged case.

**Fig. 4:**
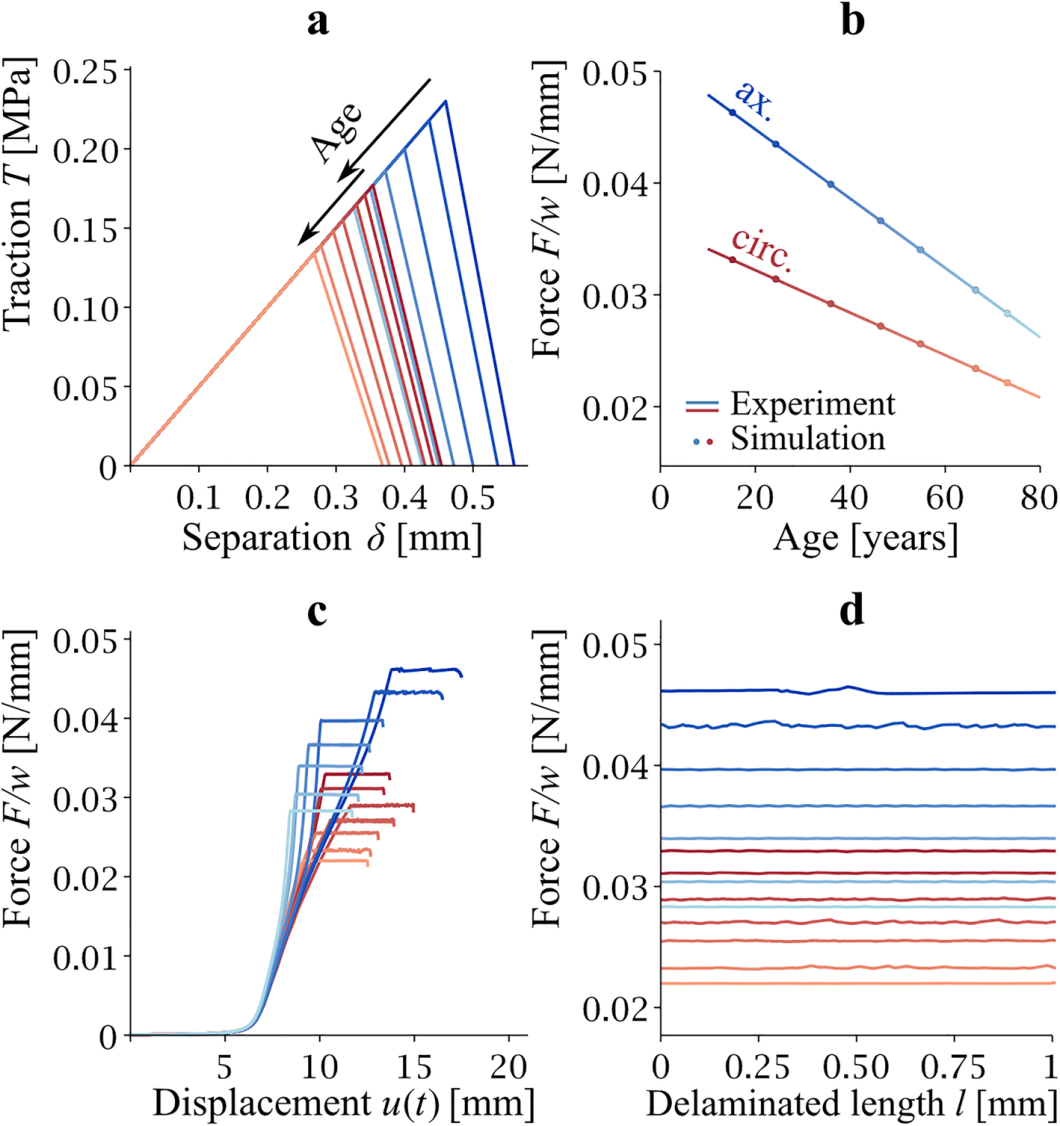
Traction–separation behavior obtained for axial (blue) and circumferential (red) orientation of delaminated strips. T-S law triangles are in (**a**). Panel **b** shows the agreement between the experimentally measured delamination strength (lines described regression equations *F*/*w* = *a*·Age + *b*) and the delamination strength predicted by the FEM model with estimated T-S law parameters. The relationship between delamination force (per width) and displacement *u* (see Figs. 1 and 2) is in panel **c** and finally panel **d** illustrates the stability of the force (per width) in FEM calculations

The results of the T-S law parameter calibration for the axially and circumferentially oriented strips are shown in Fig. 4. For the axially oriented strips, shades of blue are used, whereas for the circumferential strips, shades of red are used. The specific numerical values of *T*_*c*_ are given in Table 3. The monotonically decreasing height of the *T*_*c*_ vertices in the T-S law triangles in Fig. 4a indicates the dependence of *T*_*c*_ on age. The agreement between the experimentally measured delamination strength and the delamination force (per unit width) calculated by the FEM model using the estimated T-S law parameters (shown in Table 3) is shown in Fig. 4b. The FEM predicted values (isolated points) lie on the regression lines obtained in the regression analysis of the experiments. The agreement is very good (coefficient of determination *R*^2^ = 0.999 in both cases). The panels of Fig. 4c and d show how the value of the force reaction varies with the phase of the experiment (elastic vs. delamination).

**Table 3:** Age-dependent sets of *T*_*c*_ for axially (*T*_*c*_^ax^), circumferentially (*T*_*c*_^circ^) oriented strip and for an averaged description of delamination strength (*T*_*c*_^aver^)

Age [year]	15.3	24.4	36.0	46.5	54.9	66.5	73.2	Linear regression	*R*^2^
*T*_*c*_^ax^ [MPa]	0.230	0.218	0.200	0.186	0.175	0.163	0.155	*T*_*c*_ = − 13.03E−4·*Age* + 0.2485	0.995
*T*_*c*_^circ^ [MPa]	0.177	0.171	0.165	0.155	0.148	0.139	0.134	*T*_*c*_ = − 7.576E−4·*Age* + 0.1899	0.994
*T*_*c*_^aver^ [MPa]	0.201	0.193	0.182	0.172	0.163	0.152	0.145	*T*_*c*_ = − 9.711E−4·Age + 0.2165	0.999

Note that the full T-S law description includes K = 0.5 MPa/mm, and *δ*_f_–*δ*_c_ = 0.1 mm. The linear regression equations can be used to interpolate between age points, where FEM optimizations were performed. R^2^ denotes the coefficient of determination. Note that this R^2^ does not refer to the regression of F/w-age dependence but to T_c_-age dependence

**Fig. 5:**
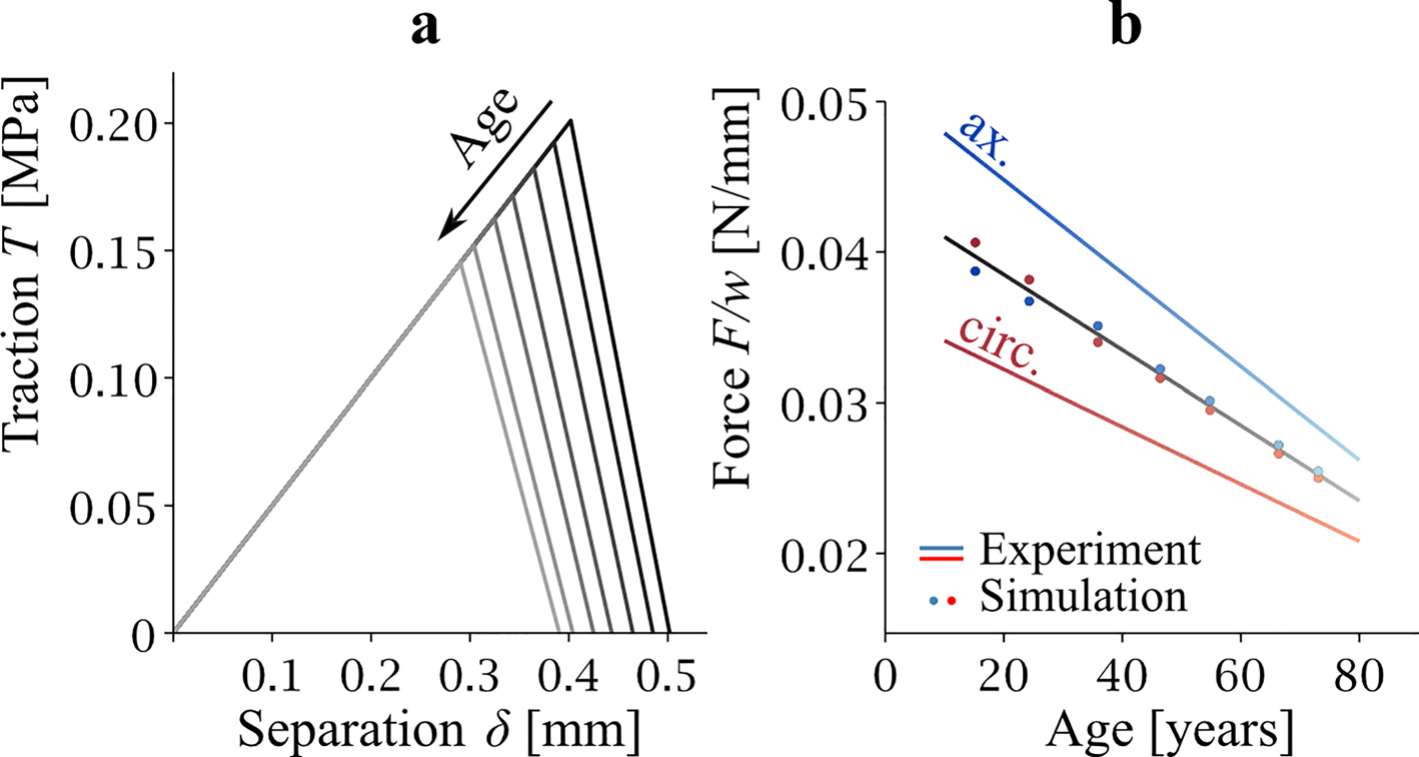
Traction–separation behavior of the averaged model. Panel **a** shows decreasing position [*δ*_*c*_,*T*_*c*_] vertices as a consequence of age-related changes to the cohesive properties. Panel **b** depicts delamination strengths at different ages calculated with FEM with average T-S law parameters. Blue and red lines are regression lines for *F*/*w* known from the experiments. Blue and red points are values of *F*/*w* calculated with the average T-S law model. The averages between blue and red points at every age were connected to the line that lies in the middle (gold)

The results for the averaged model are shown in Fig. 5 and the *T*_*c*_ values for this case are shown in Table 3. The vertices [*δ*_*c*_,*T*_*c*_] in Fig. 5a again show that the critical value of the normal component of the stress vector decreases, which is a consequence of age-related changes in the delamination strength. This is then evident in Fig. 5b, which documents the comparison between the regression lines for delamination strength obtained from the experimental data (blue and red line) and the predicted delamination strength for the average of the FEM model for the axial strip and of the FEM model for the circumferential strip (average indicated with gold line, individual predictions blue and red isolated points). The averaged T-S law parameters predict delamination strength in the middle between axial and circumferential behavior.

For the sake of completeness, the values of the fracture energy *G*_*c*_ are also presented. The magnitude of *G*_*c*_ always corresponds to the area of the triangle expressing the T-S law. Table 4 summarizes all the investigated cases and also documents the correlation of the fracture energy with age.

**Table 4:** Age-dependent sets of energy release rates *G*_*c*_ calculated by using *K*, *T*_*c*_, *δ*_*f*_–*δ*_*c*_, and linear regressions with respect to age (Fig. 6b)

Age [year]	*G*_*c*_^aver^ [mN/mm]	*G*_*c*_^ax^ [mN/mm]	*G*_*c*_^circ^ [mN/mm]
15.3	50.5	64.4	40.2
24.4	46.9	58.4	37.8
36.0	42.2	50	35.5
46.5	38.2	43.9	31.8
54.9	34.7	39.4	29.3
66.5	30.7	34.7	26.3
73.2	28.3	31.8	24.7
Linear regression	− 0.077·Age + 11.22	− 0.113·Age + 14.33	− 0.055·Age + 8.919
*R*^2^	0.999	0.998	0.996

*R*^2^ is a coefficient of determination

**Fig. 6:**
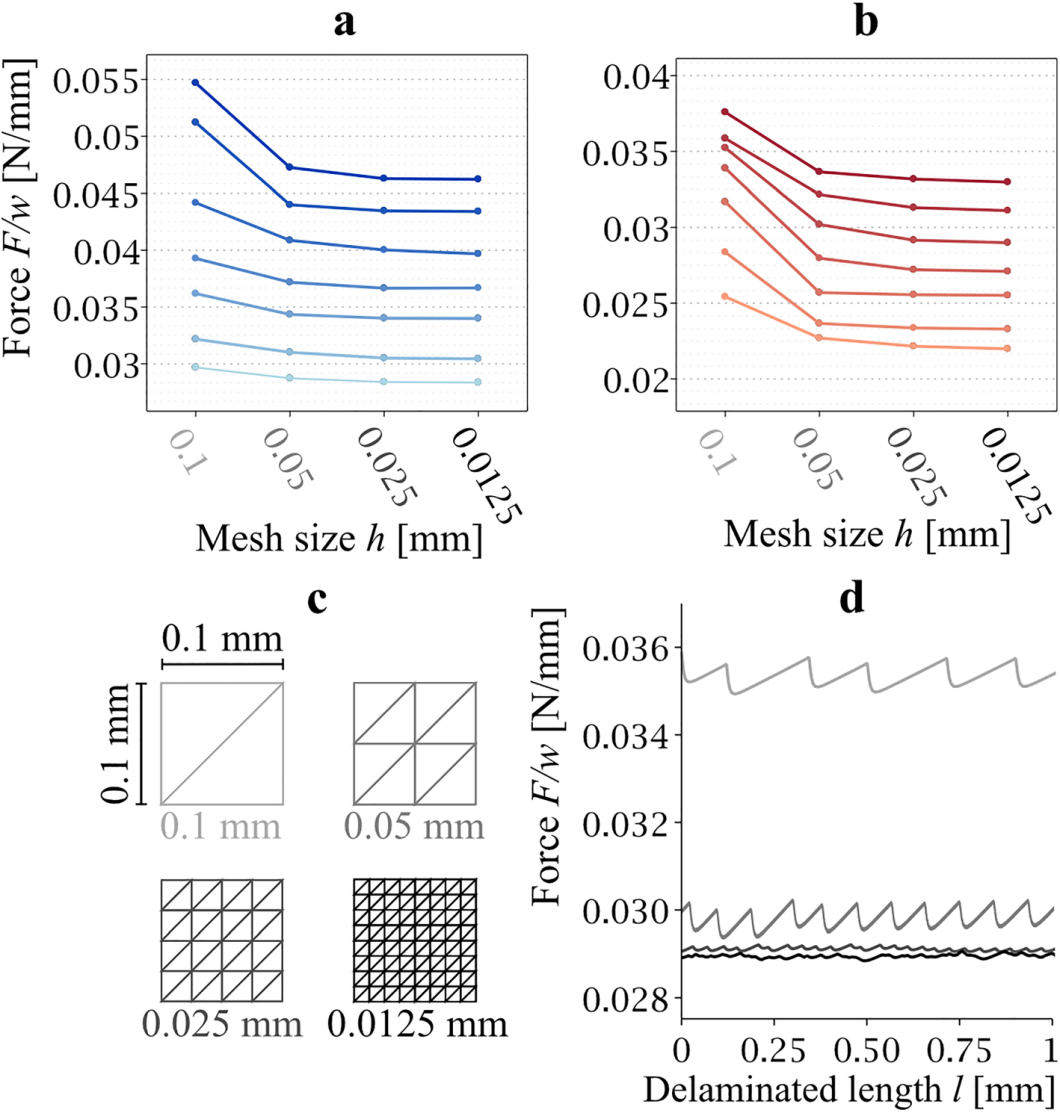
Mesh sensitivity analysis. Average values of the predicted delamination force vs. mesh size for the longitudinal strip are shown in panel **a**, and panel **b** depicts the same for the circumferential strip. Panel **c** illustrates the four sizes of elements used in the sensitivity analysis. Panel **d** gives a detailed view of the force–delamination length relationship to document the effect of the mesh size on the numerical oscillations recorded within FEM calculations. The finer the mesh is, the lower is the amplitude of the force within delamination

The results of the mesh size sensitivity analysis performed in order to verify that the estimated parameters are independent of the discretization are shown in Fig. 6. FEM meshes with edge lengths of 0.1, 0.05, 0.025, and 0.00125 mm were considered (Fig. 6c). The difference in the predicted delamination strength for the meshes with element edge lengths of 0.1 mm and 0.05 mm was greater than 10% (on average, it was 8.3% for the axially oriented strip and 16.6% for the circumferential strip, Fig. 6a, b). This deviation decreased significantly when a mesh with an edge length of 0.025 mm was used (the difference between 0.05 and 0.025 mm was 1.75% for the axial strip and 2.1% for the circumferential strip). As mentioned in methods, the resulting calibrated parameter values were obtained from simulations with a mesh size of 0.0125 mm, for which the average difference with respect to the 0.025 mm mesh was less than 1% for both orientations of the delaminated strips, therefore the results were considered to be independent of mesh size.

## Discussion

Our study provides three sets of experimentally validated parameters *T*_*c*_ that are suitable for the expression of age-related changes in the T-S law describing the cohesive behavior of the human thoracic descending aorta. A FEM model for the peeling experiment was built in Abaqus (version 2019). Predictions of delamination force were compared with delamination forces measured in our previous experiments (Horny et al. 2022). The T-S law parameter estimates were set to achieve a convergence of the FEM problem and at the same time, to ensure that the difference between the FEM-predicted delamination strength (delamination force per strip width) and the delamination strength observed in the experiments was minimal. The piecewise linear T-S law used in our study can be generally described by *K*, *δ*_*c*_, *T*_*c*_, *δ*_*f*_, and *G*_*c*_, of which only three are independent of each other. In our study, two of the three parameters, *K* and *δ*_*f*_–*δ*_*c*_, were fixed (*K* = 0.5 MPa/mm, *δ*_*f*_–*δ*_*c*_ = 0.1 mm). The value of *T*_*c*_ was then calibrated to the delamination strengths given by the regression of the experiments. The determined *T*_*c*_ values were in the interval [0.134, 0.230] MPa, with an average *T*_*c*_ equal to 0.173 MPa.

As shown above, *δ*_*f*_–*δ*_*c*_ and *K* were kept constant throughout the analysis. The main reason for this was that we experienced significant convergence difficulties during the first phase of the study when the FEM model was being tuned. These difficulties could theoretically have multiple sources. One of them could have been the constitutive model for the bulk material. However, there are not many experimentally validated parameter sets of nonlinear anisotropic constitutive models for the thoracic aorta available in the literature that account for aging, so we chose to continue to using the data from Jadidi et al. (2020). Meanwhile, additional FEM analyses conducted in the initial phase of our study did not show that the behavior of the numerical calculations changed after geometry or mesh modifications. The use of artificial damping did not lead to a significant improvement either. Finally, it was decided to consider *K* and *δ*_*f*_–*δ*_*c*_ as constant.

The reason for calibration against *T*_*c*_ rather than deformation quantities such as *δ*_*c*_ and *δ*_*f*_, and stiffness *K* is that in peeling experiments the force required for delamination is the primarily measured quantity. The delamination force is also well interpretable with respect to an onset of crack propagation which is easily, and with minimal error, identifiable on *F*-*u* recordings of peeling experiments. This is in contrast to, for example, to the cohesive bond stiffness *K*. On the other hand, it should be noted that very recent studies by Wang et al. (2021a, b) and Donahue et al. (2024) use a statistical analysis of *F*-*u* experimental recordings to estimate *K* and the separation parameters. Unfortunately, this promising approach cannot be adopted by our study, because it relies on experiments published in Horny et al. (2022), where these data are not available. In Horný et al. (2022), aging is studied in terms of delamination strength and not the age-related variability of the oscillations on the *F*-*u* recordings acquired during delamination.

In Fracture Mechanics, it is also common to use the fracture energy *G*_*c*_ to characterize the loss of cohesion during crack propagation (Irwin and Wells 1965; Zehnder 2012). In the context of peeling experiments, *G*_*c*_ has been expressed several times as *G*_*c*_ = (*W*_external_ − *W*_elastic_)/*L* = (2*Fl*/*w*—*F*Δ*l*/*w*)/*L*; Sommer et al. (2008), Tong et al. (2011). Here *L* is the length of the strip that delaminates, *l* is the length of the strip after the elastic stretching achieved before the onset of delamination, *w* is the width of the delaminated strip, and finally *F* is the delamination force. The resulting expression is *G*_*c*_ = *F*/*w*(2 + Δ*l*/*L*). Thus, in theory it is possible to use the *G*_*c*_ estimated from experiments to reduce the number of the unknown parameters of the T-S law. Unfortunately, this data is not available in Horny et al. (2022). However, even if it were available, it is not certain that it would be a suitable tool for studying age-related changes in cohesive behavior. Needleless to say, if a study works with fixed sample geometries, then the only sources of variability in *G*_*c*_ are *F*/*w* and Δ*l*/*L*. It is clear that the quantity Δ*l*/*L* contributes to the energy from the stretching of the T arms of the sample, i.e. it primarily carries information about the age-dependent elastic behavior of the bulk material. However, this information is already contained in the data for the *W* parameters in our model (the combination of Eq. 1 and the data from Table 2). It brings us back to *F*/*w* as the quantity on which our approach is based.

The last decade has seen an increasing number of studies dealing with the experimental determination of the delamination strength of the human aorta and other arteries (Sommer et al. 2008; Tong et al. 2011, 2014; Pasta et al. 2012; Kozuń 2016; Kozuń et al. 2018; Myneni et al. 2020; Horný et al. 2022). These studies have revealed the association between delamination strength and the position of the artery in the circulatory system (Tong et al. 2011; Myneni et al. 2020; Horný et al. 2022; Sokolis and Papadodima 2022a, b; Ríos-Ruiz et al. 2022), between delamination strength and the direction of the crack tip propagation (Sommer et al. 2008; Tong et al. 2011; Kozuń et al. 2016; Horný et al. 2022), between delamination strength and various types of disease (Pasta et al. 2012; Angouras et al. 2019; Chung et al. 2020; Kozuń et al. 2018; Wang et al. 2021b; Tong et al. 2023; Donahue et al. 2024), and finally, and most importantly for our present study, between delamination strength and age (Horný et al. 2022).

As somewhat different situation can be seen for papers providing experimentally validated material parameters for mathematical models of aortic wall cohesion. These works are less frequent and authors also use various concepts to introduce a mathematical description of the cohesion and its failure (Gasser and Holzapfel 2006; Ferrara and Pandolfi 2010; Merei et al. 2017; Leng et al. 2018; Wang et al. 2018, 2021a; Miao et al. 2020; FitzGibbon and McGarry 2021; Donahue et al. 2024). For example, Ríos-Ruiz et al. (2022) adopted the same T-S law. In their FEM calibration of the peeling tests, they arrived at the following estimates of the T-S law parameters: *K* = 10 MPa/mm, *δ*_*c*_ = 0.019 mm, *T*_*c*_ = 0.185 MPa, *δ*_*f*_ = 0.086 mm, and *G*_*c*_ = 8 mN/mm for an aortic strip oriented in the axial direction, and *K* = 8 MPa/mm, *δ*_*c*_ = 0.02 mm, *T*_*c*_ = 0.160 MPa, *δ*_*f*_ = 0.063 mm, and *G*_*c*_ = 5 mN/mm for the strip, where the crack tip was propagated in the circumferential direction. Their estimates of *T*_*c*_ fall within the interval [0.134, 0.230] MPa that was found for *T*_*c*_ in our study. On the other hand, the results differ in *K* by one order of the magnitude (0.5 MPa/mm vs. from 8 to 10 MPa/mm). Differences in these parameters may be affected by the different sources of tissue (human vs. porcine) and also by the exact position of the crack within the artery thickness, as well as by the heterogeneity of the aorta along its circumference (Sokolis and Papadodima 2022a, b; Xuan et al. 2023) and even along its axis, even though one takes samples from the same anatomical location (the descending part of the thoracic aorta can be longer than 30 cm).

Ferrara and Pandolfi (2010) also found *T*_*c*_ to be in order of tenths of MPa (*T*_*c*_ = 0.14 MPa, with *G*_*c*_ = 49 mN/mm which also corresponds well with our results). Studies by Leng et al. identified *T*_*c*_ = 0.05 − 0.2 MPa (with mean *G*_*c*_ = 10 mN/mm, mouse tissue, 2015), *T*_*c*_ = 0.42 MPa (2016, with *G*_*c*_ = 230 mN/mm and *K* = 10 MPa/mm, mouse tissue), and finally *T*_*c*_ = 0.44 MPa (2018, with *G*_*c*_ = 186 mN/mm and *K* = 1 MPa/mm, again mouse tissue). Wang et al. (2021a) found *T*_*c*_ = 0.06 MPa for porcine aortic media in the circumferential direction, and 0.095 MPa in the longitudinal direction (other parameters were found to be *G*_*c*_ = 0.106 mN/mm, *K* = 0.75 MPa/mm, *δ*_*f*_ = 3.55 mm, and *δ*_*c*_ = 0.08 mm for the circumferential direction, and *G*_*c*_ = 0.183 mN/mm, *K* = 1.19 MPa/mm, *δ*_*f*_ = 3.87 mm, and *δ*_*c*_ = 0.08 mm for the longitudinal direction). The results from Wang et al. (2021a) suggest somewhat different numbers than in the abovementioned studies. This again could be attributed to differences in the source tissue, material heterogeneity or to a dependence on the state of health (in the case of human case) but it is worth noting that Wang et al. (2021a) employed an analysis of oscillations on the *F*-*u* signal recorded during delamination, which suggests that future studies should evaluate this effect. As we mentioned above, we currently do not have enough data to do this in the present study.

In contrast to Ríos-Ruiz et al. (2022), Leng et al. (2015, 2016, 2018), and for example Wang et al. (2021a, b) our study is population-based. The data used to describe the elastic behavior of the aorta were adopted from Jadidi et al. (2020) and represent population representatives. These were combined with the results of our previous study (Horný et al. 2022) dealing with the delamination strength and were adopted in the form of an age-dependent regression model, i.e., again population-averaged values. Although population averages may be distant from one particular observation, especially when considering individual health conditions, if patient-specific simulation is not the goal, they represent the most appropriate way to see what is happening in the population. Thus, for expressing the age dependence of the stress at damage initiation, *T*_*c*_, which was our main goal here, population averaging seems instead to be a meaningful approach.

On the other hand we should mention one simplification that perhaps limits our results, which is the constant value of *K*. Since, *K* can be interpreted as a stiffness of the contact bond, although we do not have data to prove it, it seems unlikely that it will remain constant during aging. This hypothesis can be derived from our common experience with age-related changes in the elastic properties of the bulk material (Valenta et al. 2002, Horný et al. 2012, 2013, 2014a, b). However, before we include *K* among the free parameters to be identified in the regression model, it would be useful to expand the number of experimentally identified variables in the T-S law whose age dependence is determined by means other than FEM-*F*/*w* optimization. However, this approach is not available to us at this time, so we have decided to move the determination of the age dependence of *K* to a future study.

Another obvious limitation to the use of our results is that cohesive contact, as used, does not account for the anisotropy of the delamination strength. This is a limitation that arises from the chosen tool for the FEM model. Abaqus does not allow for an anisotropic cohesive contact. Therefore, the resulting parameters are divided into three sets. The set for axial and circumferential strips can be used in the modeling if the direction of crack propagation is known a priori. If the crack propagation direction is not known, the results from the averaged model can still be used. However, their use will introduce some error due to the neglecting of anisotropy.

## Conclusion

Our study was aimed at finding estimates of the T-S law parameters that would be validated against delamination experiments of human aortic strips. Since, the biomechanics of human arteries is significantly dependent on the age of the individual, the same can be expected not only for the material parameters of bulk constitutive models, but also for parameters expressing tissue cohesive properties. Using FEM model calibrated to the delamination strengths expressed by the regression of 246 delamination experiments with strips of the human thoracic descending aorta, the T-S law parameters for seven values of age were found. It was shown that the stress at damage initiation correlates with age similarly to the experimentally observed delamination strengths, which significantly decrease with age.

## Data Availability

No datasets were generated or analysed during the current study.

## References

[CR1] ABAQUS (2019) Analysis user’s manual, Version 2019. Dassault Systemes Simulia, Inc.

[CR2] Amabili M, Arena GO, Balasubramanian P, Breslavsky ID, Cartier R, Ferrari G, Holzapfel GA, Kassab A, Mongrain R (2020) Biomechanical characterization of a chronic type a dissected human aorta. J Biomech 110:109978. 10.1016/j.jbiomech.2020.109978 32827785 10.1016/j.jbiomech.2020.109978

[CR3] Angouras DC, Kritharis EP, Sokolis DP (2019) Regional distribution of delamination strength in ascending thoracic aortic aneurysms. J Mech Behav Biomed Mater 98:58–70. 10.1016/j.jmbbm.2019.06.001 31200336 10.1016/j.jmbbm.2019.06.001

[CR4] Belytschko T, Black T (1999) Elastic crack growth in nite elements with minimal remeshing. Int J Numer Meth Eng 45(5):601–620

[CR5] Belytschko T, Gracie R, Ventura G (2009) A review of extended/generalized fnite element methods for material modeling. Modell Simul Mater Sci Eng 17(4):043001

[CR6] Carson MW, Roach MR (1990) The strength of the aortic media and its role in the propagation of aortic dissection. J Biomech 23(6):579–588. 10.1016/0021-9290(90)90050-d 2341419 10.1016/0021-9290(90)90050-d

[CR7] Chung JC-Y, Wong E, Tang M, Eliathamby D, Forbes TL, Butany J, Simmons CA, Ouzounian M (2020) Biomechanics of aortic dissection: a comparison of aortas associated with bicuspid and tricuspid aortic valves. J Am Heart Assoc. 10.1161/jaha.120.016715 10.1161/JAHA.120.016715 PMC7792273 32750292

[CR8] Clark JM, Glagov S (1985) Transmural organization of the arterial media. The lamellar unit revisited. Arterioscler Off J Am Heart Assoc 5(1):19–34. 10.1161/01.atv.5.1.19 10.1161/01.atv.5.1.19 3966906

[CR9] Donahue CL, Badal RM, Younger TS et al (2024) Atherosclerotic calcifications have a local effect on the peel behavior of human aortic media. J Biomech Eng. 10.1115/1.4064682 10.1115/1.4064682 PMC10983699 38329432

[CR10] Ferrara A, Pandolfi A (2010) A numerical study of arterial media dissection processes. Int J Fract 166(1–2):21–33. 10.1007/s10704-010-9480-y

[CR11] FitzGibbon B, McGarry P (2021) Development of a test method to investigate mode II fracture and dissection of arteries. Acta Biomater 121:444–460. 10.1016/j.actbio.2020.11.023 33227484 10.1016/j.actbio.2020.11.023

[CR12] Forsell C, Gasser TC (2011) Numerical simulation of the failure of ventricular tissue due to deep penetration: the impact of constitutive properties. J Biomech 44(1):45–51. 10.1016/j.jbiomech.2010.08.022 20825943 10.1016/j.jbiomech.2010.08.022

[CR13] Fung YC (1993) Biomechanics: mechanical properties of living tissues, 2nd edn. Springer Science + Business Media, New York

[CR14] Fung YC, Fronek K, Patitucci P (1979) Pseudoelasticity of arteries and the choice of its mathematical expression. Am J Physiol Heart Circ Physiol 237:H620–H631. 10.1152/ajpheart.1979.237.5.H620 10.1152/ajpheart.1979.237.5.H620 495769

[CR15] Gasser TC, Holzapfel GA (2003) Geometrically non-linear and consistently linearized embedded strong discontinuity models for 3D problems with an application to the dissection analysis of soft biological tissues. Comput Methods Appl Mech Eng 192(47–48):5059–5098. 10.1016/j.cma.2003.06.001

[CR16] Gasser TC, Holzapfel GA (2006) Modeling the propagation of arterial dissection. Eur J Mech A Solids 25(4):617–633. 10.1016/j.euromechsol.2006.05.004

[CR17] Gasser TC, Ogden RW, Holzapfel GA (2006) Hyperelastic modelling of arterial layers with distributed collagen fibre orientations. J R Soc Interface 3(6):15–35. 10.1098/rsif.2005.0073 16849214 10.1098/rsif.2005.0073 PMC1618483

[CR18] Holzapfel GA (2000) Nonlinear solid mechanics: a continuum approach for engineering. Wiley, Chichester

[CR19] Holzapfel GA, Ogden RW (2010) Constitutive modelling of arteries. Proc R Soc Math Phys Eng Sci 466(2118):1551–1597. 10.1098/rspa.2010.0058

[CR20] Holzapfel GA, Gasser TC, Ogden RW (2000) A New constitutive framework for arterial wall mechanics and a comparative study of material models. J Elast 61(1/3):1–48. 10.1023/a:1010835316564

[CR21] Holzapfel GA, Niestrawska JA, Ogden RW et al (2015) Modelling non-symmetric collagen fibre dispersion in arterial walls. J R Soc Interface. 10.1098/rsif.2015.0188 10.1098/rsif.2015.0188 PMC4424700 25878125

[CR22] Horny L, Adamek T, Chlup H, Zitny R (2012) Age estimation based on a combined arteriosclerotic index. Int J Legal Med 126:321–326. 10.1007/s00414-011-0653-7 22160294 10.1007/s00414-011-0653-7

[CR23] Horny L, Adamek T, Zitny R (2013) Age-related changes in longitudinal prestress in human abdominal aorta. Arch Appl Mech 83:875–888. 10.1007/s00419-012-0723-4

[CR24] Horný L, Netušil M, Daniel M (2014a) Limiting extensibility constitutive model with distributed fibre orientations and ageing of abdominal aorta. J Mech Behav Biomed Mater 38:39–51. 10.1016/j.jmbbm.2014.05.021 25016175 10.1016/j.jmbbm.2014.05.021

[CR25] Horný L, Netušil M, Voňavková T (2014b) Axial prestretch and circumferential distensibility in biomechanics of abdominal aorta. Biomech Model Mechanobiol 13:783–799. 10.1007/s10237-013-0534-8 24136338 10.1007/s10237-013-0534-8

[CR26] Horný L, Roubalová L, Kronek J, Chlup H, Adámek T, Blanková A, Petřivý Z, Suchý T, Tichý P (2022) Correlation between age, location, orientation, loading velocity and delamination strength in the human aorta. J Mech Behav Biomed Mater 133:105340. 10.1016/j.jmbbm.2022.105340 35785636 10.1016/j.jmbbm.2022.105340

[CR27] Irwin G, Wells A (1965) A continuum-mechanics view of crack propagation. Metall Rev 10(1):223–270

[CR28] Itskov M (2019) Tensor algebra and tensor analysis for engineers (with applications to continuum mechanics), 5th edn. Springer, Cham

[CR29] Jadidi M, Habibnezhad M, Anttila E, Maleckis K, Desyatova A, MacTaggart J, Kamenskiy A (2020) Mechanical and structural changes in human thoracic aortas with age. Acta Biomater 103:172–188. 10.1016/j.actbio.2019.12.024 31877371 10.1016/j.actbio.2019.12.024 PMC6982607

[CR30] Kassab GS (2007) Design of coronary circulation: a minimum energy hypothesis. Comput Methods Appl Mech Eng 196(31–32):3033–3042. 10.1016/j.cma.2006.09.024

[CR31] Kozuń M (2016) Delamination properties of the human thoracic arterial wall with early stage of atherosclerosis lesions. J Theor Appl Mech. 10.15632/jtam-pl.54.1.229

[CR32] Kozuń M, Kobielarz M, Chwiłkowska A, Pezowicz C (2018) The impact of development of atherosclerosis on delamination resistance of the thoracic aortic wall. J Mech Behav Biomed Mater 79:292–300. 10.1016/j.jmbbm.2018.01.009 29353772 10.1016/j.jmbbm.2018.01.009

[CR33] Labrosse MR, Gerson ER, Veinot JP, Beller CJ (2013) Mechanical characterization of human aortas from pressurization testing and a paradigm shift for circumferential residual stress. J Mech Behav Biomed Mater 17:44–55. 10.1016/j.jmbbm.2012.08.004 23127625 10.1016/j.jmbbm.2012.08.004

[CR34] Leng X, Chen X, Deng X, Sutton MA, Lessner SM (2015) Modeling of experimental atherosclerotic plaque delamination. Ann Biomed Eng 43(12):2838–2851. 10.1007/s10439-015-1357-9 26101030 10.1007/s10439-015-1357-9

[CR35] Leng X, Davis LA, Deng X, Sutton MA, Lessner SM (2016) Numerical modeling of experimental human fibrous cap delamination. J Mech Behav Biomed Mater 59:322–336. 10.1016/j.jmbbm.2016.02.011 26897094 10.1016/j.jmbbm.2016.02.011 PMC5079437

[CR36] Leng X, Zhou B, Deng X, Davis L, Lessner SM, Sutton MA, Shazly T (2018) Experimental and numerical studies of two arterial wall delamination modes. J Mech Behav Biomed Mater 77:321–330. 10.1016/j.jmbbm.2017.09.025 28963936 10.1016/j.jmbbm.2017.09.025

[CR37] MacLean NF, Dudek NL, Roach MR (1999) The role of radial elastic properties in the development of aortic dissections. J Vasc Surg 29(4):703–710. 10.1016/s0741-5214(99)70317-4 10194499 10.1016/s0741-5214(99)70317-4

[CR38] Merei B, Badel P, Davis L, Sutton MA, Avril S, Lessner SM (2017) Atherosclerotic plaque delamination: experiments and 2D finite element model to simulate plaque peeling in two strains of transgenic mice. J Mech Behav Biomed Mater 67:19–30. 10.1016/j.jmbbm.2016.12.001 27988441 10.1016/j.jmbbm.2016.12.001

[CR39] Miao T, Tian L, Leng X, Miao Z, Wang J, Xu C, Liu L (2020) A comparative study of cohesive zone models for predicting delamination fracture behaviors of arterial wall. Open Phys 18(1):467–477. 10.1515/phys-2020-0134

[CR40] Mohammadi S (2008) Extended nite element method: for fracture analysis of structures. John Wiley & Sons, Hoboken

[CR41] Myneni M, Rao A, Jiang M, Moreno MR, Rajagopal KR, Benjamin CC (2020) Segmental variations in the peel characteristics of the porcine thoracic aorta. Ann Biomed Eng 48(6):1751–1767. 10.1007/s10439-020-02489-x 32152801 10.1007/s10439-020-02489-x

[CR42] Noble C, van der Sluis O, Voncken RMJ, Burke O, Franklin SE, Lewis R, Taylor ZA (2017) Simulation of arterial dissection by a penetrating external body using cohesive zone modelling. J Mech Behav Biomed Mater 71:95–105. 10.1016/j.jmbbm.2017.03.004 28284843 10.1016/j.jmbbm.2017.03.004

[CR43] Pal S, Tsamis A, Pasta S et al (2014) A mechanistic model on the role of “radially-running” collagen fibers on dissection properties of human ascending thoracic aorta. J Biomech 47:981–988. 10.1016/j.jbiomech.2014.01.005 24484644 10.1016/j.jbiomech.2014.01.005 PMC4082402

[CR44] Pasta S, Phillippi JA, Gleason TG, Vorp DA (2012) Effect of aneurysm on the mechanical dissection properties of the human ascending thoracic aorta. J Thorac Cardiovasc Surg 143(2):460–467. 10.1016/j.jtcvs.2011.07.058 21868041 10.1016/j.jtcvs.2011.07.058 PMC8084112

[CR45] Prêtre R, Von Segesser LK (1997) Aortic dissection. Lancet 349(9063):1461–1464. 10.1016/s0140-6736(96)09372-5 9164331 10.1016/S0140-6736(96)09372-5

[CR46] Prokop EK, Palmer RF, Wheat MW Jr (1970) Hydrodynamic forces in dissecting aneurysms. Circ Res 27(1):121–127. 10.1161/01.res.27.1.121 10.1161/01.res.27.1.121 5424561

[CR47] Ríos-Ruiz I, Cilla M, Martínez MA, Peña E (2021) Methodology to calibrate the dissection properties of aorta layers from two sets of experimental measurements. Mathematics 9(14):1593. 10.3390/math9141593

[CR48] Ríos-Ruiz I, Martínez MÁ, Peña E (2022) Is location a significant parameter in the layer dependent dissection properties of the aorta? Biomech Model Mechanobiol 21(6):1887–1901. 10.1007/s10237-022-01627-9 36057051 10.1007/s10237-022-01627-9 PMC9700619

[CR49] Roach MR, Song SH (1994) Variations in strength of the porcine aorta as a function of location. Clin Invest Med 17(4):308–318 7982294

[CR50] Sherifova S, Holzapfel GA (2019) Biomechanics of aortic wall failure with a focus on dissection and aneurysm: a review. Acta Biomater 99:1–17. 10.1016/j.actbio.2019.08.017 31419563 10.1016/j.actbio.2019.08.017 PMC6851434

[CR51] Sherifova S, Holzapfel GA (2020) Biochemomechanics of the thoracic aorta in health and disease. Progress Biomed Eng 2(3):032002. 10.1088/2516-1091/ab9a29 10.1088/2516-1091/ab9a29 41559965

[CR52] Sokolis DP, Papadodima SA (2022a) Regional delamination strength in the human aorta underlies the anatomical localization of the dissection channel. J Biomech 141:111174. 10.1016/j.jbiomech.2022.111174 35701262 10.1016/j.jbiomech.2022.111174

[CR53] Sokolis DP, Papadodima SA (2022) Regional delamination strength in the human aorta underlies the anatomical localization of the dissection channel. J Biomech. 10.1016/j.jbiomech.2022.111174 10.1016/j.jbiomech.2022.111174 35701262

[CR54] Sommer G, Gasser TC, Regitnig P, Auer M, Holzapfel GA (2008) Dissection properties of the human aortic media: an experimental study. J Biomech Eng. 10.1115/1.2898733 10.1115/1.2898733 18412494

[CR55] Spencer AJM (1982) Deformation of fiber-reinforced materials. Oxford University Press, Oxford

[CR56] Takamizawa K, Hayashi K (1987) Strain energy density function and uniform strain hypothesis for arterial mechanics. J Biomech 20:7–17. 10.1016/0021-9290(87)90262-4 3558431 10.1016/0021-9290(87)90262-4

[CR57] Tanaka H, Okada K, Kawanishi Y, Matsumori M, Okita Y (2009) Clinical significance of anastomotic leak in ascending aortic replacement for acute aortic dissection. Interact Cardiovasc Thorac Surg 9(2):209–212. 10.1510/icvts.2008.201558 19454413 10.1510/icvts.2008.201558

[CR58] Thubrikar MJ (2007) Vascular mechanics and pathology. Springer, US

[CR59] Tiessen IM, Roach MR (1993) Factors in the initiation and propagation of aortic dissections in human autopsy aortas. J Biomech Eng 115(1):123–125. 10.1115/1.2895461 8445891 10.1115/1.2895461

[CR60] Tong J, Sommer G, Regitnig P, Holzapfel GA (2011) Dissection properties and mechanical strength of tissue components in human carotid bifurcations. Ann Biomed Eng 39(6):1703–1719. 10.1007/s10439-011-0264-y 21308483 10.1007/s10439-011-0264-y

[CR61] Tong J, Cohnert T, Regitnig P, Kohlbacher J, Birner-Gruenberger R, Schriefl AJ, Sommer G, Holzapfel GA (2014) Variations of dissection properties and mass fractions with thrombus age in human abdominal aortic aneurysms. J Biomech 47(1):14–23. 10.1016/j.jbiomech.2013.10.027 24309621 10.1016/j.jbiomech.2013.10.027

[CR62] Tong J, Cheng Y, Holzapfel GA (2016) Mechanical assessment of arterial dissection in health and disease: advancements and challenges. J Biomech 49(12):2366–2373. 10.1016/j.jbiomech.2016.02.009 26948576 10.1016/j.jbiomech.2016.02.009

[CR63] Tong J, Xin YF, Zhang Z, Xu X, Li T (2023) Effect of hypertension on the delamination and tensile strength of ascending thoracic aortic aneurysm with a focus on right lateral region. J Biomech 154:111615. 10.1016/j.jbiomech.2023.111615 37178496 10.1016/j.jbiomech.2023.111615

[CR64] Truesdell C, Noll W (2004) The non-linear field theories of mechanics. Springer, Berlin Heidelberg

[CR65] Tsamis A, Phillippi JA, Koch RG et al (2013) Fiber micro-architecture in the longitudinal-radial and circumferential-radial planes of ascending thoracic aortic aneurysm media. J Biomech 46:2787–2794. 10.1016/j.jbiomech.2013.09.003 24075403 10.1016/j.jbiomech.2013.09.003 PMC3898198

[CR66] Valenta J, Vitek K, Cihak R, Konvickova S, Sochor M, Horny L (2002) Age related constitutive laws and stress distribution in human main coronary arteries with reference to residual strain. Bio-Med Mater Eng 12(2):121–134 12122236

[CR67] van Baardwijk C, Roach MR (1987) Factors in the propagation of aortic dissections in canine thoracic aortas. J Biomech 20(1):67–73. 10.1016/0021-9290(87)90268-5 3558430 10.1016/0021-9290(87)90268-5

[CR68] Wang L, Hill NA, Roper SM, Luo X (2018) Modelling peeling- and pressure-driven propagation of arterial dissection. J Eng Math 109(1):227–238. 10.1007/s10665-017-9948-0 31258175 10.1007/s10665-017-9948-0 PMC6566306

[CR69] Wang R, Yu X, Gkousioudi A, Zhang Y (2021a) Effect of glycation on interlamellar bonding of arterial elastin. Exp Mech 61:81–94. 10.1007/s11340-020-00644-y 33583947 10.1007/s11340-020-00644-y PMC7880226

[CR70] Wang R, Yu X, Zhang Y (2021b) Mechanical and structural contributions of elastin and collagen fibers to interlamellar bonding in the arterial wall. Biomech Model Mechanobiol 20:93–106. 10.1007/s10237-020-01370-z 32705413 10.1007/s10237-020-01370-z PMC7855057

[CR71] Wang X, Carpenter HJ, Ghayesh MH, Kotousov A, Zander AC, Amabili M, Psaltis PJ (2023) A review on the biomechanical behaviour of the aorta. J Mech Behav Biomed Mater 144:105922. 10.1016/j.jmbbm.2023.105922 37320894 10.1016/j.jmbbm.2023.105922

[CR72] Weisbecker H, Unterberger MJ, Holzapfel GA (2015) Constitutive modelling of arteries considering fibre recruitment and three-dimensional fibre distribution. J R Soc Interface. 10.1098/rsif.2015.0111 10.1098/rsif.2015.0111 PMC4387538 25788541

[CR73] Wells GN, de Borst R, Sluys LJ (2002) A consistent geometrically non-linear approach for delamination. Int J Numer Meth Eng 54(9):1333–1357

[CR74] Wolinsky H, Glagov S (1967) A lamellar unit of aortic medial structure and function in mammals. Circ Res 20(1):99–111. 10.1161/01.res.20.1.99 4959753 10.1161/01.res.20.1.99

[CR75] Xuan Y, Wang Z, Guccione JM (2023) Regional and directional delamination properties of healthy human ascending aorta and sinotubular junction. J Mech Behav Biomed Mater. 10.1016/j.jmbbm.2022.105603 10.1016/j.jmbbm.2022.105603 36512974

[CR76] Yu X, Suki B, Zhang Y (2020) Avalanches and power law behavior in aortic dissection propagation. Sci Adv. 10.1126/sciadv.aaz1173 10.1126/sciadv.aaz1173 PMC7244314 32494736

[CR77] Zehnder AT (2012) Fracture mechniacs. In: Pfeiffer F, Wriggers P (eds) Lecture notes in applied and computational mechanics. Springer Science+Business Media, Cham

